# Epoxyeicosatrienoic Acid Analog Decreases Renal Fibrosis by Reducing Epithelial-to-Mesenchymal Transition

**DOI:** 10.3389/fphar.2017.00406

**Published:** 2017-06-30

**Authors:** Melissa Skibba, Md. Abdul Hye Khan, Lauren L. Kolb, Michael M. Yeboah, John R. Falck, Radhika Amaradhi, John D. Imig

**Affiliations:** ^1^Department of Pharmacology and Toxicology, The Medical College of Wisconsin, MilwaukeeWI, United States; ^2^Department of Medicine, The Medical College of Wisconsin, MilwaukeeWI, United States; ^3^Department of Biochemistry, UT Southwestern Medical Center, DallasTX, United States

**Keywords:** EET analog, EMT, UUO, Snail1, ZEB1

## Abstract

Renal fibrosis, which is a critical pathophysiological event in chronic kidney diseases, is associated with renal epithelial-to-mesenchymal transition (EMT). Epoxyeicosatrienoic acids (EETs) are Cyp epoxygenase arachidonic acid metabolites that demonstrate biological actions that result in kidney protection. Herein, we investigated the ability of 14,15-EET and its synthetic analog, EET-A, to reduce kidney fibrosis induced by unilateral ureter obstruction (UUO). C57/BL6 male mice underwent sham or UUO surgical procedures and were treated with 14,15-EET or EET-A in osmotic pump (i.p.) for 10 days following UUO surgery. UUO mice demonstrated renal fibrosis with an 80% higher kidney-collagen positive area and 70% higher α-smooth muscle actin (SMA) positive renal areas compared to the sham group. As a measure of collagen content, kidney hydroxyproline content was also higher in UUO (6.4 ± 0.5 μg/10 mg) compared to sham group (2.5 ± 0.1 μg/10 mg). Along with marked renal fibrosis, UUO mice had reduced renal expression of EET producing Cyp epoxygenase enzymes. Endogenous 14,15-EET or EET-A demonstrated anti-fibrotic action in UUO by reducing kidney-collagen positive area (50–60%), hydroxyproline content (50%), and renal α-SMA positive area (85%). In UUO mice, renal expression of EMT inducers, Snail1 and ZEB1 were higher compared to sham group. Accordingly, renal epithelial marker E-cadherin expression was reduced and mesenchymal marker expression was elevated in the UUO compared to sham mice. Interestingly, EET-A reduced EMT in UUO mice by deceasing renal Snail1 and ZEB1 expression. EET-A treatment also opposed the decrease in renal E-cadherin expression and markedly reduced several prominent renal mesenchymal/myofibroblast markers in UUO mice. Overall, our results demonstrate that EET-A is a novel anti-fibrotic agent that reduces renal fibrosis by decreasing renal EMT.

## Introduction

Renal tubulointerstitial injury is associated with chronic kidney diseases and is linked to fibrotic pathway activation and ultimate progression to end-stage renal failure. It is proposed that renal fibrosis is the strongest pathological predictor for chronic kidney disease progression and the clinical outcome (Liu, 2011). Although there are controversies, emerging evidence indicates that renal tubular epithelial-to-mesenchymal transition (EMT) is an important event in the pathophysiology of renal fibrosis (Kalluri and Neilson, 2003; Kizu et al., 2009; Loeffler and Wolf, 2014, 2015; Nakasatomi et al., 2015). Indeed, *in vitro*, *in vivo*, and renal biopsy findings indicate that myofibroblasts are formed in diseased kidneys from the tubular epithelium through the tubular EMT route (Rastaldi, 2006; Flier et al., 2010). It is, therefore, conceivable that developing a novel approach that will inhibit myofibroblast accumulation and thereby targeting EMT would be an effective therapeutic option to treat renal fibrosis, and preserve renal function in chronic kidney diseases.

Regarding development of a novel treatment approach for renal fibrosis, use of epoxyeicosatrienoic acids (EETs), the Cyp epoxygenase metabolites, is potentially promising. EETs are endothelium derived hyperpolarizing factors that have diverse physiological actions including vasodilatory, anti-hypertensive, anti-inflammatory, and anti-apoptotic actions that would decrease kidney damage (Imig and Hammock, 2009; Imig, 2012). Nevertheless, endogenously produced EETs are chemically and metabolically labile which critically limit their therapeutic potential (Falck et al., 2009; Imig and Falck, 2009). To circumvent this limitation with endogenous EETs, several synthetic EET analogs have been developed. EET analogs have biological activity and many structural features required for stability and bioavailability (Imig et al., 2010; Batchu et al., 2011). We developed EET-A, a synthetic analog of 14,15-EET, which is the most abundant EET in the kidney (Karara et al., 1990). In designing EET-A the carboxyl group in the EET pharmacophore was modified by conjugation with an aspartic acid moiety. In recent studies, we demonstrated that EET-A possesses robust kidney protective actions in several pre-clinical kidney disease models with varied etiologies (Hye Khan et al., 2013a, 2014, 2016).

With this background, the current study examined EET-A and 14,15-EET for anti-fibrotic and kidney protective effects in kidney fibrosis induced by unilateral ureter obstruction (UUO). We demonstrate comparable kidney protective effects for EET-A and the endogenous 14,15-EET. Further studies were carried out with EET-A and demonstrated its ability to reduce kidney fibrosis by decreasing renal EMT.

## Materials and Methods

### Chemicals

14,15-EET was purchased from Cayman Chemical Company (Ann Arbor, MI, United States) while its analog, EET-A, was designed and synthesized in the laboratory of John R. Falck (Department of Biochemistry, University of Texas Southwestern Medical Center, Dallas, TX, United States). All other chemicals used in this study were purchased from Sigma–Aldrich (St. Louis, MO, United States) unless mentioned otherwise.

### Animal Experiments

This study was approved and conducted according to guidelines of the Institutional Animal Care and Use Committee, Medical College of Wisconsin. The Biomedical Resource Center at the Medical College of Wisconsin housed animals with free access to water and food and a 12/12 h light-dark cycle. Male C57Bl/6J mice (8- to 10-weeks-old) were purchased from Jackson Laboratories, Bar Harbor, ME, United States. Mice were administered 2.0% isoflurane to induce anesthesia prior to UUO surgery. UUO surgery was conducted with by obstructing the left ureter proximal to the renal pelvis using a 6–0 silk tie (Kim et al., 2015; Sharma et al., 2016). Mice with sham surgery went through the same procedure as the UUO mice except that the ureter was not ligated. UUO-mice were randomly assigned into three groups, vehicle-UUO group (*n* = 10) received the vehicle (25% DMSO in PEG-400) and two treatment groups received 14,15-EET (10 mg/kg/d, *n* = 6) or EET-A (10 mg/kg/d, *n* = 6). Doses are based on previous experimental studies demonstrating that appropriate EET-A plasma concentrations were achieved (Imig et al., 2010; Hye Khan et al., 2013b, 2014; Sharma et al., 2016) Vehicle or treatments (14,15-EET and EET-A) were administered for a 10-day experimental period via intra-peritoneal osmotic pump (ALZET^®^ osmotic pump, DURECT Corporation, Cupertino, CA, United States). Plasma and kidney samples were collected 10 days following surgery. Kidney samples for histological studies were fixed in 10% buffered formalin and stored at room temperature. Tissue samples for biochemical analysis were snap-frozen in liquid nitrogen and stored at -80°C.

### Biochemical Analysis

Blood urea nitrogen (BUN) was measured spectrophotometrically using a commercial kit (BioAssay Systems, Hayward, CA, United States). Kidney hydroxyproline levels were measured using a previously described method (Sharma et al., 2016). In brief, a kidney section was homogenized in 10N HCl and hydrolyzed. The hydrolysate was then incubated in Chloramine T reagent [42 mM sodium acetate, 0.84% chloramines-T, 2.6 mM citric acid, and 39.5% isopropanol (pH 6.0)]. Lastly, DMAB reagent [15% 4-(dimethylamino)benzaldehyde in isopropanol/perchloric acid (2:1 vol/vol)] was added to each sample and hydroxyproline levels were determined at 550 nm.

### Real-Time PCR Analysis

Renal mRNA expression for the epithelial marker E-cadherin (*Cdh1*); mesenchymal markers α-SMA (*Acta2*), fibroblast specific protein-1 (FSP-1) (*Atl1*), fibronectin (*Fn1*), and desmin (*Des*); and EMT gene transcription regulators Snail1 (*Snai1*) and ZEB1 (*Zeb1*) was determined by conducting Real Time-PCR (RT-PCR) analysis. RNeasy Mini Kit (QIAGEN, Valencia, CA, United States) was used according to the manufacturer’s protocol and messenger RNA (mRNA) was prepared from each sample homogenate. The mRNA samples were quantified spectrophotometrically and 1μg of total RNA was reverse-transcribed to cDNA using iScript^TM^ Select cDNA Synthesis Kit (Bio-Rad, Hercules, CA, United States). Gene expression was quantified by iScript One-Step RT-PCR Kit with SYBR green using the MyiQ^TM^ Single Color Real-Time PCR Detection System (Bio-Rad Laboratories, Hercules, CA, United States). Dissociation curve analysis was accomplished with iQ5 Optical System Software, Version 2.1 (Bio-Rad Laboratories, Hercules, CA, United States), and each amplified sample analyzed for homogeneity. Denaturation was done at 95°C for 2 min followed by 40 cycles conducted at 95°C for 10 s and at 60°C for 30 s. All samples were run in triplicate and fold change in gene expression compared to controls determined by comparative threshold cycle (C_t_) method. Target gene expression levels were determined by normalizing *C*_t_ values to two housekeeping genes. Statistical analyses were carried out using six samples from each experimental group and comparing to the control group.

### Western Blot Analysis

Frozen kidney samples were homogenized in RIPA buffer containing a protease and phosphatase inhibitor cocktail (Sigma, St. Louis, MO, United States). Kidney homogenate protein concentrations were quantified with a bicinchoninic acid (BCA) assay (Pierce Biotechnology, Rockford, IL, United States). Kidney sample proteins (60 μg/lane) were separated by SDS–polyacrylamide gel electrophoresis followed by transfer to a polyvinylidene difluoride membrane (Bio-Rad, Hercules, CA, United States). Non-specific binding to the membrane was blocked with a solution containing 5% milk in TBS plus 0.1% Tween-20 (TBST). The membrane was then incubated with primary antibodies: collagen 3A1 (Santa Cruz Biotechnology, 1:1000) and β-tubulin and E-cadherin (Cell Signaling, 1:1000) or ZEB1 (Novus Biologicals, 1:1000) overnight at 4°C. ZEB1 was probed after stripping the original membrane with Restore Western Blot Stripping Buffer (Thermo Fisher Scientific, Waltham, MA United States). For determination of protein levels blots were washed with TBST followed by incubating with horseradish peroxidase conjugated goat anti-rabbit or anti-mouse IgG (Cell Signaling, 1:5000) for 1 h at room temperature. Next, protein bands were detected using SuperSignal West Femto Maximum Sensitivity Substrate (Thermo Fisher). Blot images were collected on a BioRad Chemidoc system and protein expression levels quantitated via densitometry using ImageQuant TL Version 8.1 software.

### Histopathology

Renal tissues were fixed in 10% formalin, sectioned a 5 μm thickness, mounted on slides, and stained with Periodic Acid-Schiff (PAS) (Acros Organics, Fair Lawn, NJ, United States) or Picrosirius Red (PSR) (Alfa Aesar, Tewksbury, MA, United States) for histological examination at a 200× magnification using NIS Elements AR version 3.0 imaging software (Nikon instruments Inc., Melville, NY, United States). PAS-stained tissue was evaluated for tubular injury whilst PSR was used to determine collagen-positive renal interstitial fibrosis levels. Histopathological changes were scored as published previously and scores presented as a percentage area-fraction relative to the total area analyzed (Sharma et al., 2016). Tubular injury (PAS-cast areas) and interstitial fibrosis (PSR-collagen positive areas) scoring was performed in a blinded fashion by two observers.

### Immunohistopathological Analysis

Kidney histological slides were deparaffinized and re-hydrated followed by overnight incubation with anti-α smooth muscle actin (α-SMA) antibody (1:100, Santa Cruz Biotechnology, Dallas, TX, United States). On the second day, the slides were washed and exposed to biotinylated rat anti-mouse secondary antibody (1:200) for 1 h. α-SMA positive was developed with avidin-biotinylated HRP complex (Vectastain ABC Elite kit, Vector Laboratories, Burlingame, CA, United States) followed by counterstaining with hematoxylin. Stained histological sections were visualized at 400× magnification with a light microscope and analyzed using Nikon NIS Elements Software (Nikon Instruments Inc., Melville, NY, United States). Kidney α-SMA positive areas expressed as the percentage area were verified by two observers.

### Statistical Analysis

All data are expressed as mean ± SEM GraphPad Prism^®^ Version 4.0 software was utilized to conduct a one-way ANOVA followed by Tukey’s *post hoc* test to establish statistical significance between groups (GraphPad Software Inc, La Jolla, CA, United States). Two-tailed unpaired Student’s *t*-test was applied to determine statistical significance between groups. A *P* < 0.05 was deemed significant.

## Results

### Renal Expression of Cyp Epoxygenases Were Lower in UUO

Interestingly, we demonstrated a marked 80% lower renal mRNA expression for the major murine epoxygenase producing Cyp (Cyp2c44 and Cyp2j5) enzymes in the UUO renal fibrosis compared to sham mice. The relative fold changes (2^-ΔΔCt^) in UUO kidney mRNA expression for Cyp2c44 and Cyp2j5 were 0.23 ± 0.22 and 0.14 ± 0.16, respectively, compared to 1.02 ± 0.37 and 1.03 ± 0.13 in sham mice.

### 14,15-EET and EET-A Minimized Kidney Injury in UUO

**FIGURE 1:**
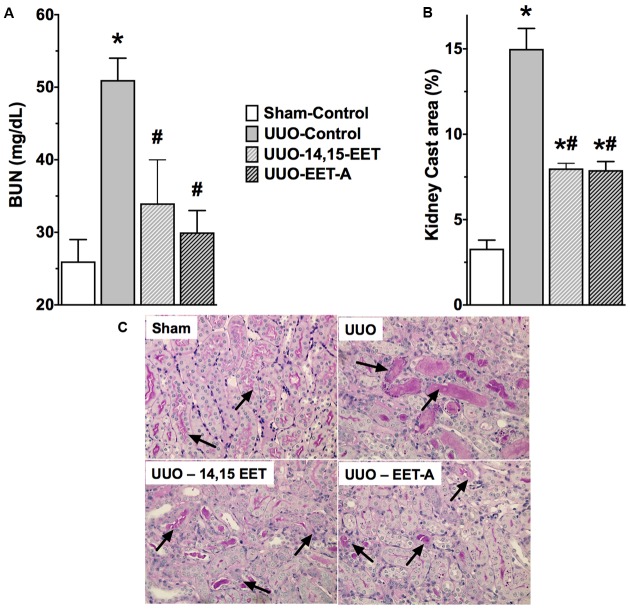
An endogenous EET, 14,15-EET and a synthetic EET analog EET-A reduced kidney injury in a UUO renal fibrosis model. **(A)** Blood urea nitrogen and **(B)** tubular cast formation in different experimental groups. **(C)** A representative photomicrographs showing tubular cast formation (black arrows) in different experimental groups. All data are expressed as Mean ± SEM, ^∗^*P* < 0.05 vs. Sham-Vehicle, ^#^*P* < 0.05 vs. UUO-Vehicle, *n* = 6.

UUO mice had higher BUN levels (51 ± 3 mg/dL) compared to sham mice (26 ± 3 mg/dL) that indicates marked renal injury in UUO renal fibrotic mice (**Figure 1**). Kidney injury in UUO mice was further demonstrated by a fivefold increase in renal cast area compared to sham mice (**Figure 1**). In mice subjected to UUO, 14,15-EET or EET-A treatment comparably decreased BUN levels and reduced renal tubular cast formation by nearly 50%.

### 14,15-EET and EET-A Attenuate Progressive Renal Fibrosis in UUO

**FIGURE 2:**
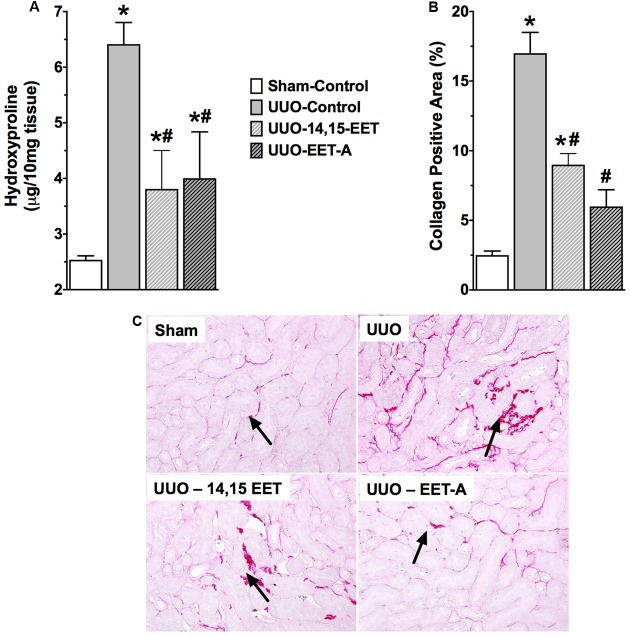
An endogenous EET, 14,15-EET and a synthetic EET analog EET-A reduced kidney fibrosis in UUO by reducing kidney **(A)** hydroxyproline content, and **(B)** collagen positive area in the kidney. **(C)** Representative photomicrographs showing renal interstitial fibrosis as collagen deposition (black arrows). All data are expressed as Mean ± SEM, ^∗^*P* < 0.05 vs. Sham-Vehicle, ^#^*P* < 0.05 vs. UUO-Vehicle, *n* = 6.

Unilateral ureter obstruction renal fibrotic mice demonstrated marked renal fibrosis with higher renal collagen content (6.4 ± 0.5 μg/10 mg tissue) compared to sham mice (2.5 ± 0.1 μg/10 mg tissue) (**Figure 2**). Renal fibrosis in UUO mice was more evident from an eightfold increase in collagen positive fibrotic area compared to sham mice (**Figure 2**). 14,15-EET or EET-A administration prevented collagen accumulation in UUO mice, and reduced collagen content and renal collagen positive area by 50% compared to vehicle treated UUO mice.

### EET-A Prevents Renal Epithelial-to-Mesenchymal Transition (EMT) in UUO Mice

**FIGURE 3:**
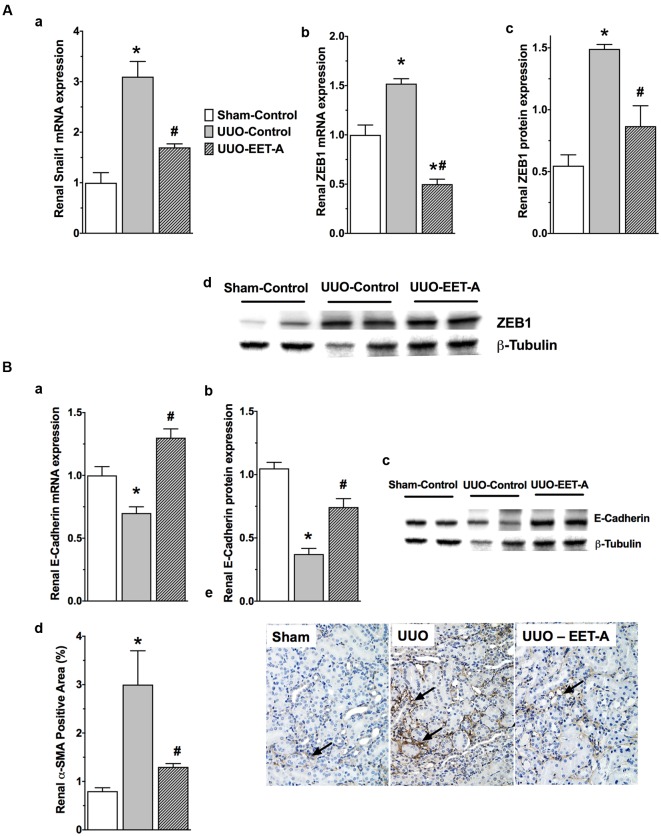
**(A)** The synthetic EET analog EET-A reduced renal EMT in the kidney of UUO mice by reducing renal expression of the EMT inducers Snail1 (a) and ZEB1 (b–d). All data are expressed as Mean ± SEM, ^∗^*P* < 0.05 vs. Sham-Vehicle, ^#^*P* < 0.05 vs. UUO-Vehicle, *n* = 6. **(B)** The synthetic EET analog EET-A prevented the decrease in renal expression of E-cadherin (a–c) and markedly attenuated α-SMA expression (d,e) in the kidney of UUO mice. All data are expressed as Mean ± SEM, ^∗^*P* < 0.05 vs. Sham-Vehicle, ^#^*P* < 0.05 vs. UUO-Vehicle, *n* = 6.

**FIGURE 4:**
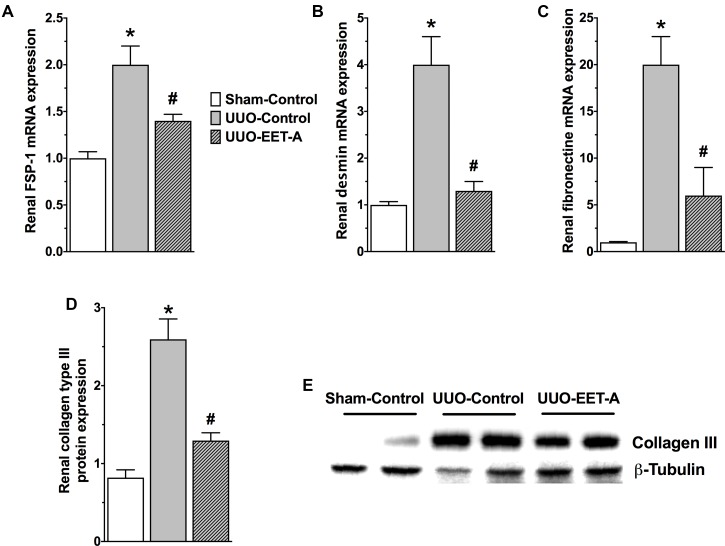
EET-A reduced renal EMT by decreasing renal mRNA expressions of prominent myofibroblast marker fibroblast specific protein-1 (FSP-1) **(A)** and several fibrotic cytoskeletal and extracellular matrix protein markers like desmin, fibronectin, and collagen type III **(B–E)** in the kidney of UUO mice. All data are expressed as Mean ± SEM, ^∗^*P* < 0.05 vs. Sham-Vehicle, ^#^*P* < 0.05 vs. UUO-Vehicle, *n* = 6.

Unilateral ureter obstruction renal fibrotic mice developed renal EMT and had an elevated renal expression of EMT regulator transcription factors Snail1 and ZEB1 compared to sham mice (**Figure 3A**). Along with an elevated renal Snail1 and ZEB1 expression, UUO mice displayed reduced renal expression of the prominent epithelial marker E-cadherin that indicates EMT induction (**Figure 3B**). As strong evidence for renal EMT, UUO mice also demonstrated a marked 70% higher renal protein expression of the mesenchymal/myofibroblast marker α-SMA (**Figure 3B**). Interestingly, EET-A markedly prevented the elevation in renal Snail1 expression in UUO mice and brought Snail1 expression levels down to a level comparable to that in sham mice. EET-A also markedly reduced renal expression of another EMT inducer ZEB1 in UUO mice compared to vehicle treated UUO mice (**Figure 3A**). Accordingly, EET-A prevented the reduction in renal expression of the epithelial marker E-cadherin and markedly reduced expression of the mesenchymal marker α-SMA in UUO mice. Indeed, EET-A treatment reduced renal α-SMA expression in UUO mice to a level comparable to that in sham mice (**Figure 3B**). In addition to a strong effect on the expression of α-SMA in UUO mice, EET-A treatment reduced renal expression of several other markers for myofibroblast (FSP-1) and fibrotic cytoskeletal protein markers (fibronectin and desmin). EET-A treatment also resulted in marked reduction in renal matrix protein collagen type III expression in UUO mice (**Figure 4**). Taken together, these findings demonstrate that EET-A has a strong inhibitory effect on renal EMT in UUO mice.

## Discussion

Chronic kidney disease is rapidly increasing worldwide, and this is to a large degree due to an increase in the incidence of hypertension, obesity, diabetes, and other cardiovascular diseases (Harris and Neilson, 2006). Renal fibrosis is a common finding in various forms of chronic kidney diseases with diverse etiologies. Renal fibrotic progression is considered as a major pathological process leading to a gradual loss of renal function in chronic kidney diseases (Zeisberg and Neilson, 2010). Current therapeutic options for kidney fibrosis are limited and often are not fully effective. This limitation in the availability and usefulness of reliable and effective therapeutic options warrants development of novel renal anti-fibrotic approaches. To this end, in the present study, we examined the anti-fibrotic effectiveness of an EET analog, EET-A, and an endogenous EET, 14,15-EET, in a UUO renal fibrosis model. UUO is a well-established and widely used model for investigating renal fibrotic mechanisms and therapeutic strategies (Chevalier et al., 2009).

In the present study, our rational approach was to examine the anti-fibrotic ability for 14,14-EET and EET-A analog treatment in UUO mice came from the interesting finding that UUO mice had markedly lower renal mRNA expression of EET producing Cyp epoxygenase Cyp2c44 and Cyp2j5 enzymes. Renal expression of EET producing Cyp enzymes has been reported in several pathological conditions (Wang et al., 2003; Zhao et al., 2003; Capdevila et al., 2014; Hye Khan et al., 2016). It has been demonstrated that in a diet-induced and salt-sensitive hypertension that a decrease renal Cyp2c23 expression is associated with renal dysfunction (Wang et al., 2003; Zhao et al., 2003). In a recent study, we demonstrated that development of radiation-induced renal injury is associated with lower Cyp2c23 and Cyp2c11 levels in the kidney (Hye Khan et al., 2016). Although the exact cellular signal or molecular pathway leading to decreased renal Cyp2c expression is unknown, these findings demonstrate that decreased Cyp2C expression and EET levels contribute to renal diseases.

Our finding that there was reduced renal Cyp epoxygenase expression in the UUO renal fibrotic mice provided evidence for a contribution decreased EET levels in the renal pathology. This also suggested the possibility that a novel EET-based approach could circumvent this problem in renal fibrosis. Moreover, considering the strong kidney protective actions of EETs in different pathological models, we propose that exogenous administration of EETs or EET analogs could provide beneficial anti-fibrotic effects in UUO renal fibrotic mice.

Unilateral ureter obstruction mice develop renal dysfunctions that include renal tubular injury with marked interstitial fibrosis. Our current findings are in accord to data obtained in several earlier studies demonstrating this renal pathology in UUO models (Kawaoka et al., 2016; Wu et al., 2016). Interestingly, EET-A, a synthetic mimetic of 14,15-EET, or 14,15-EET prevented renal dysfunction, tubular injury and fibrosis in UUO mice. Renal protective actions for EETs have been reported in many pathologies. It was shown that an increase in EET levels by inhibiting soluble epoxide hydrolase (sEH), an enzyme that metabolize endogenous EETs to their less active vicinal diols, protected the kidney and reduced renal fibrosis (Imig, 2012). It has been suggested that anti-fibrotic and kidney protective actions for EETs are associated with their ability to reduce blood pressure or blood glucose. However, there are experimental studies that demonstrate kidney protective actions for EETs during sEH inhibition in kidney injury models that are not associated with change in blood pressure or glycemic status (Parrish et al., 2009; Kim et al., 2014, 2015). These findings have provided evidence that EET kidney protective actions can occur independent of reduced blood pressure or blood glucose. Inhibition of sEH either pharmacologically or genetically resulted in an increase in endogenous EET bioavailability and a reduction in UUO induced renal fibrosis (Kim et al., 2014, 2015). Likewise, pharmacological sEH inhibition decreased cisplatin-induced nephropathy (Parrish et al., 2009). Overall, these findings clearly indicate an important contribution for reduced EETs to renal fibrosis and raise the possibility that an sEH inhibitory-based therapy could effectively treat renal fibrosis.

Nevertheless, there are limitations in developing the sEH inhibitory-based therapeutic approach. The sEH inhibitors result in a generalized increase in EETs and their therapeutic effectiveness depends on Cyp epoxygenase-mediated EET generation (Imig and Hammock, 2009; Imig, 2010, 2012). This significantly limits the effective use for sEH inhibitors in cardiovascular or renal therapy because many renal and cardiovascular diseases are associated with impaired EET generation (Imig and Hammock, 2009; Imig, 2010, 2012). As such, if Cyp epoxygenase mediated EET generation is impaired in a pathological condition then sEH inhibition will have a decreased ability to maximally increase EET levels. Another therapeutic limitation for sEH inhibition is that endogenously produced EETs are chemically and metabolically labile (Imig and Falck, 2009; Falck et al., 2009). With this background, attempts have been made to develop synthetic EET analogs that have chemical and structural features to increase stability and bioavailability (Sudhahar et al., 2010). The molecular target or receptor for EETs and the EET-A analog remain unknown. Evidence suggests that a G protein coupled EET receptor exist and the structure activity relationship for EET analogs has been extensively defined (Falck et al., 2009; Imig, 2010, 2012). EET analogs such as EET-A utilize identical cell-signaling mechanisms as EETs and EET antagonists block these actions (Falck et al., 2009; Sudhahar et al., 2010; Hye Khan et al., 2014). Thus, a phenotypic drug development approach has been applied to develop orally active EET analogs.

Several recently developed EET analogs have demonstrated promising kidney protective actions. Indeed, we previously demonstrated that EET analogs, including EET-A, reduced kidney fibrosis in hypertensive renal injury and in radiation nephropathy (Hye Khan et al., 2014, 2016). Nonetheless, in these studies the observed anti-fibrotic actions for EET analogs were accompanied by a reduction in blood pressure, hence, this made it difficult to ascertain an independent anti-fibrotic action for EET analogs in these disease states. In the present study, considering the anti-fibrotic action of 14,15-EET, we examined if its synthetic analog EET-A has anti-fibrotic actions in UUO mice, which is not associated with any systemic elevation in blood pressure. Interestingly, we EET-A demonstrated marked anti-fibrotic action in UUO mice and clearly indicate its potential in developing an EET-based renal anti-fibrotic agent. Indeed, there is an unmet need for innovative approaches to prevent/treat renal fibrosis because renal fibrosis has been identified as a major pathological process leading to the decline in renal function that occurs in chronic kidney diseases (Zeisberg and Neilson, 2010). In this study, we also explored the mechanism of the anti-fibrotic action for EET-A in the renal fibrosis UUO mouse model.

The mechanism of renal fibrosis is multifactorial and involves accumulation of extracellular matrix factors and loss of tubular architecture. There is evidence to support the notion that the tubular epithelial cells make an important contribution to renal fibrosis (Liu et al., 2012). A critical step in renal fibrotic pathogenesis is EMT, whereby renal tubular epithelial cells change from a mesenchymal phenotype and function into myofibroblasts (Zeisberg et al., 2002). This EMT transformation is characterized by E-cadherin loss (epithelial marker) and increased α-SMA and FSP-1 (mesenchymal/myofibroblast marker) (Zeisberg et al., 2008; Cook, 2010). On the other hand, there are experimental findings indicating that activated myofibroblasts originate from multiple lineages that cast doubt on the specific contribution of EMT to renal fibrosis *in vivo*, and has led many to speculate on the exact cell types involved in renal fibrosis (Loeffler and Wolf, 2015). Indeed, the exact origin for myofibroblasts in the fibrotic kidney remains controversial (Loeffler and Wolf, 2014). A crucial advance in our understanding of renal fibrosis is that multiple cell types are responsible for the accumulation and remodeling of extracellular matrix components (Simonson, 2007). Experimental findings demonstrate that myofibroblasts can be derived from bone marrow, tubular epithelium, vascular endothelium, and pericytes (Kizu et al., 2009).

Despite the controversy on the origin of myofibroblasts in the kidney and subsequent renal fibrosis, several recent studies provide convincing evidence for a critical EMT contribution to the kidney fibrosis pathology. These studies have used genetic lineage tracing systems and BrdU labeling techniques for tubular epithelial cells in different *in vivo* renal disease models including UUO and provide strong evidence for the EMT contribution to kidney fibrosis (Inoue et al., 2015; Nakasatomi et al., 2015). These findings suggest that therapies that prevent tubular epithelial cells from undergoing EMT can effectively treat tubulointerstitial fibrosis. To this end, in the present study, we examined if the EET-A anti-fibrotic actions in UUO renal fibrotic mice are associated with a reduction in EMT. We demonstrated elevated renal Snail1 and ZEB1 gene expression that are critical for EMT induction in UUO mice. These transcription factors activate EMT by binding to E-box elements present in the E-cadherin promoter, suppressing synthesis of this cell–cell adhesion protein. ZEB1 also promotes EMT by repressing the expression of basement membrane components and cell polarity proteins (Batlle et al., 2000; Das et al., 2009). A critical role for these EMT inducer genes is recognized in UUO renal fibrosis (Lange-Sperandio et al., 2007; Pu et al., 2016). It is reported that in UUO renal fibrosis, Snail1 expression is elevated within 24 h, and precedes α-SMA induction in the kidney (Lange-Sperandio et al., 2007). Interestingly, in the present study EET-A reduced expression of the EMT inducer genes, Snail1 and ZEB1, prevented the decrease in E-cadherin expression, and reduced expression of mesenchymal/myofibroblast markers. Overall, we demonstrated that in a UUO model that renal fibrosis is associated with EMT. We further demonstrated that EET-A is anti-fibrotic in the kidney and markedly reduced renal EMT by decreasing the expression of two prominent EMT inducer genes Snail1 and ZEB1. Nonetheless, it is possible that EET-A has additional mechanisms that along with reducing renal EMT contribute to the decreased renal fibrosis in UUO model.

In summary, in the present study we describe anti-fibrotic actions for 14,15-EET and its synthetic analog EET-A. Most importantly, we demonstrate that the marked EET-A anti-fibrotic action is associated with its ability to reduce the occurrence of EMT in the kidney. Our findings clearly indicate a promising possibility to develop a novel EET-based therapeutic approach for renal fibrosis.

## Author Contributions

MS, MAHK, and JI conceived the study and wrote the manuscript. MS generated and interpreted gene expression and western immunoblot data. LK and MAHK performed biochemical analysis and animal experiments. MY edited the manuscript and JF and RA synthesized EET-A. All authors edited the manuscript, approved data and the final version for submission.

## Conflict of Interest Statement

JI and JF have patents that cover the composition of matter for EET-A. The other authors declare that the research was conducted in the absence of any commercial or financial relationships that could be construed as a potential conflict of interest. The reviewer IF and handling Editor declared their shared affiliation, and the handling Editor states that the process nevertheless met the standards of a fair and objective review.
